# Locally produced natural conditioners for dewatering of faecal sludge

**DOI:** 10.1080/09593330.2016.1165293

**Published:** 2016-04-18

**Authors:** Moritz Gold, Pauline Dayer, Marie Christine Amie Sene Faye, Guillaume Clair, Alsane Seck, Seydou Niang, Eberhard Morgenroth, Linda Strande

**Affiliations:** ^a^ Eawag: Swiss Federal Institute of Aquatic Science and Technology, Dübendorf, Switzerland; ^b^ ETH Zürich, Institute of Environmental Engineering, Zürich, Switzerland; ^c^ Faculty of Medicine, Pharmacy and Odontology, Department of Pharmacy, Cheikh Anta Diop University of Dakar, Dakar-Fann, Senegal; ^d^ Department of Water Engineering, Polytech Nice-Sophia, Sophia Antipolis, France; ^e^ Faculty of Sciences and Technics, Institute of Environmental Sciences (ISE), Cheikh Anta Diop University of Dakar, Dakar-Fann, Senegal; ^f^ Laboratory of Wastewater Treatment, Fundamental Institute of Black Africa (IFAN), Cheikh Anta Diop University of Dakar, Dakar-Fann, Senegal

**Keywords:** Sanitation, developing country, dewatering, coagulation, chitosan

## Abstract

In urban areas of low-income countries, treatment of faecal sludge (FS) is insufficient or non-existent. This results in large amounts of FS being dumped into the environment. Existing treatment technologies for FS, such as settling-thickening tanks and drying beds, are land intensive which is limiting in urban areas. Enhanced settling and dewatering by conditioning was evaluated in order to reduce the treatment footprint (or increase treatment capacity). Conventional wastewater conditioners, such as commercially available lime and polymers, are expensive, and commonly rely on complex supply chains for use in low-income countries. Therefore, the treatment performance of five conditioners which could be produced locally was evaluated: *Moringa oleifera* seeds and press cake, *Jatropha curcas* seeds, *Jatropha Calotropis* leaves and chitosan. *M. oleifera* seeds and press cake, and chitosan improved settling and dewatering and had a similar performance compared to lime and polymers. Optimal dosages were 400–500 kg *M. oleifera*/t TS, 300–800 kg lime/t TS and 25–50 kg polymer solution/t TS. In comparison, chitosan required 1.5–3.75 kg/t TS. These dosages are comparable to those recommended for wastewater (sludge). The results indicate that conditioning of FS can reduce total suspended solids (TSS) in the effluent of settling-thickening tanks by 22–81% and reduce dewatering time with drying beds by 59–97%. This means that the area of drying beds could be reduced by 59–97% with end-use as soil conditioner, or 9–26% as solid fuel. Least expensive options and availability will depend on the local context. In Dakar, Senegal, chitosan produced from shrimp waste appears to be most promising.

**Abbreviations**
COD: chemical oxygen demand
EC: electric conductivity
FS: faecal sludge
FSM: faecal sludge management
FSTP: faecal sludge treatment plant
NH_4_-N: ammonium nitrogen
NO_3_-N: nitrate nitrogen
TS: total solids
TSS: total suspended solids
TVS: total volatile solids
SRF: specific resistance to filtration
SVI: sludge volume index

## 1. Introduction

Worldwide, the sanitation needs of 2.7 billion people are met by on-site sanitation technologies such as septic tanks and pit latrines, which are not connected to a sewer.[1] Faecal sludge (FS) is *the raw or partially digested, semisolid or slurry resulting from collection, storage or treatment of combinations of excreta and blackwater, with or without greywater* that accumulates in these technologies.[2] On-site sanitation technologies can provide sustainable and more affordable sanitation solutions for dense urban areas, if comprehensive faecal sludge management (FSM) is in place, including reliable collection, transport, treatment and safe end-use or disposal of FS.[3] However, In urban areas of low-income countries, adequate FS treatment and safe end-use or disposal is almost non-existent.[4,5] For example, in Hanoi, Vietnam, only 5% of FS is treated, resulting in the discharge of an estimated 750 m^3^/day into the environment [6,7]; in Accra, Ghana, 0% of FS is treated resulting in the discharge of 750 m^3^/day at a dumping point into the ocean; and in Dakar, Senegal, 25% of FS is treated with 6000 m^3^/day estimated to accumulate in on-site sanitation technologies.[8]

In addition, low-income countries are undergoing the fastest rates of urbanization in the world,[9] meaning that available space in urban areas for the treatment of FS is a challenge. Settling–thickening tanks and drying beds are the most common treatment technologies for solid–liquid separation and dewatering of FS,[10,11] however, they are very land intensive.[12] In addition, FS is typically >90% water, which is prohibitively expensive to transport.[13] Hence, existing treatment technologies need to be optimized to increase capacity and make treatment within urban areas feasible.

The use of commercial conditioners, such as polyelectrolytes and hydrolysed metals, to increase settling and dewatering performance is commonplace in wastewater treatment.[14] Settling and dewatering properties, and hence appropriate use of conditioners, vary between sludge types (e.g. primary, secondary or digested sludge). This is due to the degree of stabilization, which affects the content of inorganic matter, particle size and extracellular polymeric substances.[15–17] Primary wastewater sludge dewaters better than other sludge types, as dewatering performance decreases with particle size and sludge stabilization.[14,18] However, FS which is partially stabilized has poor dewatering performance (US EPA, 1984). FS is also highly variable, for example unstabilized when collected frequently from public toilets versus stabilized when septic tanks are emptied over a period of years.[19] FS characteristics are very different from wastewater sludge, with typically one to two orders of magnitude higher solid, organic and nutrient concentrations.[13,20] Hence, the transferability of the use of conditioners from wastewater sludge to FS cannot be assumed.

Commercial conditioners for treatment of wastewater sludge are expensive, and in low-income countries, relying on the import of products that are not locally available has been identified as a frequent reason for failure of treatment plants.[21] Conditioners that are produced from locally available resources could provide a more sustainable and affordable solution worldwide. Applications of conditioners from natural resources include a turbidity removal of 95% with *Moringa oleifera* seeds in industrial wastewater,[22] 98% with *Jatropha curcas* seeds in synthetic wastewater,[23] and an increase of two to three times with chitosan in dewatering of water treatment sludge.[24] These results indicate the potential use for dewatering of FS, however, based on the available literature, the use of natural conditioners for FS has not yet been reported.

The objective of this study was to identify conditioners for FS that could be produced with natural resources available in low-income countries, and to compare their performance to commercially available wastewater sludge conditioners as a metric to evaluate performance. The overall goal was to identify ways to increase settling and dewatering performance to increase treatment capacities of faecal sludge treatment plants (FSTPs), and hence reduce the required land area.

## 2. Material and methods

This research took place over a period of eight months at Cambérène Wastewater and FSTP in Dakar, Senegal. The process flow for FS treatment is bar screens, settling-thickening tanks and drying beds. The effluent from settling–thickening tanks and leachate from drying beds is co-treated with wastewater.

### 2.1. Conditioners

In this study, a conditioner was defined as a product which has the potential to increase settling and dewatering of FS. Five conditioners were selected for experiments based on the literature regarding conditioners for water and wastewater sludge, and their potential to be locally available in Dakar: *M. oleifera* seeds and press cake, *J. curcas* seeds, *Calotropis procera* leaves and chitosan.[22–26] For comparison of treatment performance, three commercially available wastewater sludge conditioners were selected: Lime and the two polymers CP314 and C2064. The origin and characteristics of the conditioners used in this study are summarized in Table 1.

**Table 1.** Origin and characteristics of conditioners used in this study [27–31].

*Name*	*M. oleifera* seeds	*M. oleifera* press cake	*J. curcas* seeds	*C. procera* leaves	Heppix A	lime ip410	CP314	C2064
*Origin*	Market in Dakar	Oil extraction company	Market in Dakar	Trees at Cambérène FSTP	BioLog Heppe, Germany	Heidelberg Cement, Germany	Flonex, Switzerland	Ensola Wassertechnik, Switzerland
*Charge*	Cationic	Cationic	–	–	Cationic	–	Cationic	Cationic
*Structure*	–	–	–	–	Linear	–	Linear	Linear

### 2.2. Preparation of conditioners

*M. oleifera* and *J. curcas* seeds were shelled and dried at 45°C for 24–48 hours.[22,25,27,28] *M. oleifera* press cake was dried at the same temperature. *C. procera* leaves were dried in the sun for one week and then dried at 45°C for one hour.[26] Dried seeds, press cake and leaves were then crushed with a household blender into a fine powder, and extracted with distilled water to produce a stock solution of 5% (wt./vol.).[22,25,27,28] *M. oleifera* and *J. curcas* were extracted for one and two minutes, respectively, in a blender, and *C. procera* by mixing for 20 minutes with a magnetic stirrer.[23,25]

Chitosan was obtained from the manufacturer as a 2% solution, which was diluted with distilled water to a 0.5% (wt./vol.) stock solution. Lime was used as received by the manufacturer in a powder form. The polymers obtained from the manufacturers were considered as 100% solution with a density of 1 kg/l and diluted to 0.5% and 1.0% (wt./vol.) stock solutions, respectively. Stock solutions were prepared daily to avoid effects due to storage.

### 2.3. Faecal sludge sampling

FS was collected for all repetitions of experiments on the first day. FS was collected from vacuum trucks while discharging at the FSTP. Samples were collected from five to seven trucks in the middle of tank discharge and were transferred immediately to the laboratory. One composite sample was prepared and stored at 8°C for a maximum of six days. Prior to use, the composite sample was homogenized.

### 2.4. Faecal sludge conditioning

FS was conditioned with a jar test device (Velp Scientifica FC6S). Conditioners were added in different dosages to 800 mL FS, and compared in parallel to a control with no conditioner. Based on a literature review and preliminary experiments with variable mixing times and speeds, 200 rpm for two minutes was selected for mixing during jar tests.[22,25] A minimum of five dosages from the stock solutions were used for conditioning of FS within the following ranges: 3.9–46.4 mL/g TS *M. oleifera* seeds; 3.8–25.1 mL/g TS *M. oleifera* press cake; 2–40 mL/g TS *J. curcas* seeds; 0.0085–14.1 mL/g TS *C. procera leaves*; 0.07–1.7 mL/g TS chitosan; 0.3–2.4 g/g TS lime; 2.9–27.7 mL/g TS CP314 and 1.3–18.5 mL/g TS C2064. Although the pH has a large influence on conditioning, its effect on FS conditioning was not investigated as part of this study, as additional treatment costs such as pH control for application in low-income countries wanted to be avoided.[14]

### 2.5. Settling experiments

Settling experiments were conducted with Imhoff cones. Conditioned FS was poured into graduated Imhoff cones and the volume of settled sludge was recorded. Following 60 minutes of settling, a representative grab sample was collected from the supernatant for analysis. Sludge Volume Index (SVI) which is correlated to total suspended solids (TSS) is the standard method to evaluate settling properties of wastewater sludge.[29] However, SVI could not be employed for FS as the settling of TSS showed a high variability and not all TSS settled out. Therefore, the settled sludge volume and TSS in the supernatant after 60 minutes were used to evaluate conditioner dosage for optimal settling. Settling experiments were replicated with different FS a minimum of three times, and up to eight times (see supplementary information).

### 2.6. Dewatering experiments

Dewatering was measured by specific resistance to filtration (SRF) according to EN 14701-2:2013.[30] 100 mL of settled sludge decanted from Imhoff cones was placed on a 90 mm Buchner funnel with a Whatman Grade 1 filter. The sludge was dewatered at a vacuum of 50 kPa while recording filtrate volume over time. If 100 mL of settled sludge was not available from Imhoff cones experiments due to poor settling, whatever volume was available was used for the SRF experiment. SRF was recorded as zero in case filtration was completed before the vacuum reached 50 kPa or filtration time was below 15 seconds. Dewatering experiments were replicated with different FS a minimum of three times, and up to eight times (see supplementary information).

### 2.7. Optimal conditioner dosage

In this study, due to the large variability of results, for a consistent method of reporting they are presented as the range of minimum and maximum observation. The increase in settling and dewatering by conditioning is expressed as percent reduction, comparing TSS in the supernatant after settling and SRF after dewatering of conditioned with unconditioned FS, according to:
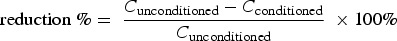
where *C* is TSS in the supernatant or SRF of unconditioned (*C*_unconditioned_) and conditioned (*C*_conditioned_) FS. In the same way, the percent increase in settled sludge volume is calculated and the sign of the result inverted. Conditioners are most effective (i.e. increase in settling and dewatering versus conditioner dosage) at low dosages. Therefore, based on the absolute performance of conditioners to increase settling and dewatering, the optimal dosage was defined as the dosage above which a consistent 75% increase in performance was measured. Results from all settling and dewatering experiments of FS in Imhoff cones and SRF experiments, as well as from bench-scale experiments in settling and dewatering columns (see below) are presented in the supplementary information.

### 2.8. Settling and dewatering columns

Four conditioners were selected for further bench-scale tests with settling and dewatering columns. Settling columns were designed to replicate treatment in settling–thickening tanks and comprising acrylic glass graduated cylinders with an inner diameter of 10 cm and a height of 100 cm. Three times conditioners were added to six 800 mL beakers of FS with the jar test device, and then poured into one settling column. Following settling, the settled sludge volume was recorded and one composite sample was taken from the supernatant for analysis. The settling velocity was calculated from the slope of the linear part of the settling curve according to Tchobanoglous et al. [14]

Filter columns comprised plastic pipes with an inner diameter of 11 cm and a height of 114 cm. Columns were filled with 10 cm coarse gravel (7–25 mm), 10 cm fine gravel (3–10 mm) and 5 cm sand (0.2–0.6 mm) to replicate drying beds. Sand was sieved and washed prior to use. The entire volume of settled sludge from the settling columns was loaded onto one dewatering column for six days. Filter loading rates were between 2.5–5.7 kg TS/m^2^ for unconditioned FS, 2.8–5.8 kg TS/m^2^ for chitosan, 9.5–13.1 kg TS/m^2^ for lime, 2.8–5.8 kg TS/m^2^ for CP314 and 2.9–6.0 kg TS/m^2^ for C2064. All leachate from dewatering columns was collected for analysis. Experiments with settling and dewatering columns were replicated with different FS three times. During replications, the same dewatering columns were used with the same conditioners.

### 2.9. Analyses

Unconditioned FS was analysed for electric conductivity (EC), pH, salinity, temperature, total solids (TS), total volatile solids (TVS), TSS, chemical oxygen demand (COD), ammonium nitrogen (NH_4_-N) and nitrate nitrogen (NO_3_-N). Supernatant from Imhoff cones and settling columns and leachate from dewatering columns were analysed for EC, pH, salinity, temperature, TS, TSS and COD. The analysis of solids parameters was based on Standard Methods.[29] TS were measured gravimetrically by drying in an oven at 105°C, and TVS at 550°C. Cellulose nitrated or glass fiber filters with a diameter of 47 mm and a pore size between 0.7 and 1.2 µm were used for TSS analysis. COD was determined with Hach vials, a Hach DRB200 heating block and a Hach DR4000v and a Dr. Lange Lasa50 spectrophotometer based on the manufacturer’s directions. EC, temperature and salinity were determined with a WTW MultiLine P4 and pH with a HANNA HI 9124 according to the manufacturer’s directions.

## 3. Results and discussion

### 3.1. Faecal sludge characteristics

Results of the physical, chemical and biochemical characteristics of FS that was collected from vacuum trucks and used in the experiments are presented in Table 2. The values and the variability are similar to those observed by other studies in Dakar.[12,31] For example, Sonko et al. [31] reported average TSS and COD concentrations of 1.3–19.9 and 1.8–21.3 g/l compared to 1.7–16.5 and 2.1–18.1 g/l in this study, respectively. A composite sample was prepared from five to seven trucks for each repetition of the experiments to reduce variability; however, variability was still high between repetitions.

**Table 2.** Physical, chemical and biochemical parameters of FS used in the experiments.

Repetition	TS (g/l)	TSS (g/l)	TVS (g/l)	COD (g/l)	NH_4_-N (mg/l)	NO_3_-N (mg/l)	pH (–)	EC (mS/cm)	Salinity (g/l)
1	19.1	16.5	11.6	18.1	421.7	61.0	7.9	3.9	2.0
2	6.8	5.1	3.9	7.7	216.0	27.6	7.8	2.7	1.3
3	9.3	7.6	6.0	13.1	576.0	38.1	7.7	6.0	3.2
4	8.9	6.3	8.7	5.4	–	–	7.9	6.2	3.3
5	11.5	9.4	7.6	10.8	–	–	7.9	4.3	2.2
6	4.9	3.5	2.8	6.2	–	–	8.0	4.5	2.4
7	9.4	6.6	5.4	6.2	–	–	7.9	3.4	1.8
8	5.1	3.7	2.7	4.9	346.0	22.1	7.9	4.1	2.1
9	5.8	3.9	3.8	4.9	526.0	26.7	7.9	5.6	3.0
10	2.9	1.7	1.3	2.4	154.4	10.7	7.8	2.6	1.2
11	6.0	4.6	3.4	3.3	–	–	8.4	4.6	2.4
12	13.1	11.6	7.8	17.5	–	–	8.3	5.3	2.8
13	16.5	16.0	–	2.1	–	–	8.0	2.5	1.2
Average	9.2	7.4	5.4	8.2	373.3	31.0	7.9	4.3	2.2

### 3.2. Settling

Settling results of unconditioned FS were highly variable. TSS and COD in the supernatant of unconditioned FS after settling ranged from 0.7 to 3.6 g/l and from 1.6 to 4.2 g/l, respectively. TSS of the supernatant collected from the Imhoff cones after 60 minutes had TSS concentrations 78–95% of unconditioned FS prior to settling.

**Figure 1.**
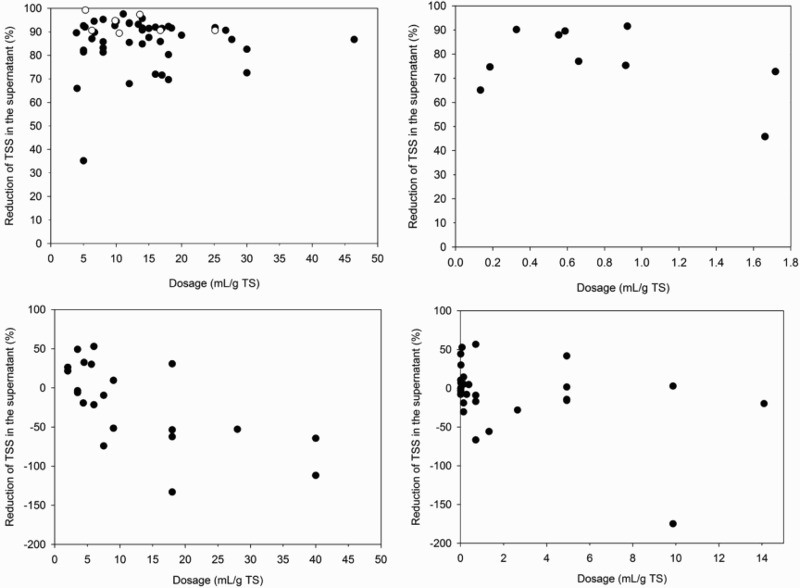
Reduction of TSS in the supernatant of FS conditioned with *M. oleifera* seeds (top, left, filled circles) and press cake (top, left, open circles) (top, left), chitosan (top, right), *J. curcas* seeds (bottom, left) and *C. procera* leaves (bottom, right).

**Figure 2.**
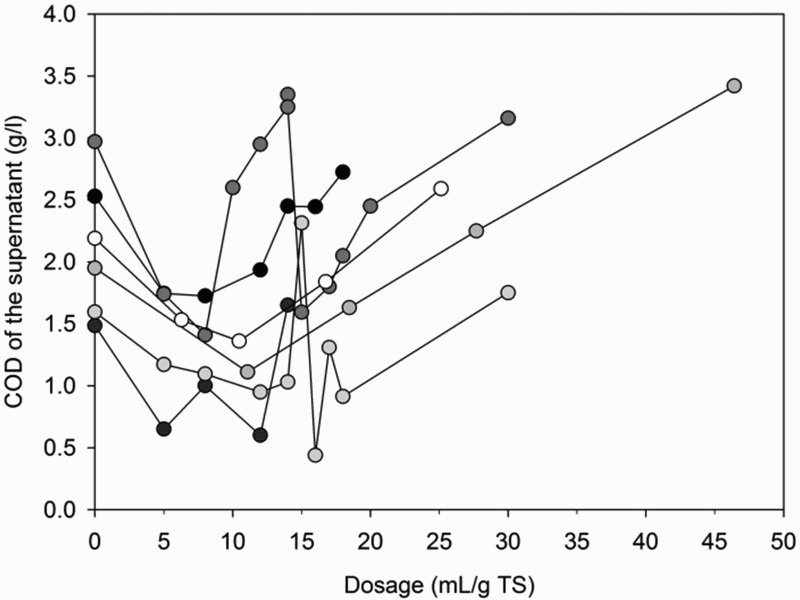
COD of the supernatant of FS conditioned with *M. oleifera* seeds in five repetitions.

Figure 1 shows conditioning results with *M. oleifera* seeds and press cake, chitosan, *J. curcas* seeds and *C. procera* leaves. Conditioning with *M. oleifera* seeds reduced TSS in the supernatant to 0.02–0.5 g/l with lower TSS concentrations at higher dosages. As shown in Figure 1, this corresponds to reductions in the range of 35–98%. Settling was optimal at dosages of around 6–8 mL/g TS with reductions in TSS in the supernatant of 81–95%. The settled sludge volume increased with higher dosage by 52–310%. Reasons for this are likely the increased settling of TSS, addition of TSS by insoluble *M. oleifera* seed particles and an increase in floc size compared to unconditioned FS [22,27]. As shown in Figure 2, conditioning with *M. oleifera* seeds reduced COD of the supernatant at dosages below 6–8 mL/g TS, whereas they increased at higher dosages.[22] also observed an increase in COD of the supernatant for conditioning of domestic and industrial wastewaters with *M. oleifera* seeds. This can be explained by the high COD concentration of the *M. oleifera* stock solution which outweighs the reduction in COD by settling of TSS.[32] reported a COD of 15 g/l for a *M. oleifera* stock solution with the same concentration as used in this study. Figure 1 shows that conditioning with *M. oleifera* press cake produced comparable results to seeds. This result is in line with the results of [33] who observed similar settling when comparing wastewater sludge conditioned with *M. oleifera* seeds and press cake.

Conditioning with chitosan reduced TSS in the supernatant to 0.07–0.45 g/l with lower TSS concentrations at higher dosages. As shown in Figure 1, this corresponds to reductions in the range of 46–92%. Settling was optimal at dosages of around 0.3–0.6 mL/g TS with reductions in TSS in the supernatant of 88–90%. The volume of settled sludge increased with dosage to 6–78%. COD of the supernatant was only measured for one repetition and reduced from 1.9 g/l to 0.5–0.8 g/l with lower concentrations at higher dosages. At dosages exceeding 1.5 mL/g TS, TSS and COD of the supernatant increased compared to lower dosages. This indicates overdosing which decreased settling and increased COD of the supernatant and the settled sludge volume compared to results at optimal dosage. According to Christensen et al. [34], Nguyen et al. [35] and Sanin et al. [36] this can be explained by saturation or charge reversal of colloids, disaggregation and dispersion of flocs and increase in supernatant viscosity.

In contrast to *M. oleifera* seeds and press cake, and chitosan, conditioning results with *J. curcas* seeds and *C. procera* leaves were not consistent, for example, higher dosages of conditioners resulted in both an increase and decrease in TSS (see Figure 1) and COD in the supernatant and settled sludge volume (detailed results presented in supplementary information). Concentrations of TSS in the supernatant were between 0.5 and 2.5 g/l for *J. curcas seeds* and 0.6 and 3.3 g/l for *C. procera leaves*. This corresponds to reductions in TSS between −133 and 53% for *J. curcas seeds* and −175 and 57% for *C. procera leaves*. Increased dosages of conditioner also in general increased COD of the supernatant. The volume of settled sludge increased with higher dosages in the range of 30–155% for *J. curcas* seeds and −11–48% for *C. procera leaves*. The poor performance of *J. curcas* could be attributed to the neutral pH of FS, as [23] observed that *J. curcas* seeds performance was optimal at pH less than 3 or greater than 11, with reduced performance of up to 50% at neutral pH for turbidity removal in wastewater.[26] also reported *C. procera* leaves were not as effective for water treatment, with 26% turbidity removal compared to 85% for *M. oleifera* seeds.

In comparison to conditioners which could be produced locally, conditioning with lime and commercially available polymers reduced TSS in the supernatant to 0.08–0.4 g/l for lime, 0.1–0.4 g/l for CP314 and 0.2–0.7 g/l for C2064 with lower TSS concentrations at higher dosages. This corresponds to reductions in the range of 63–93% for lime, 89–99% for CP314 and 56–98% for C2064. Settling was optimal at dosages of around 0.7–0.8 g/g TS for lime, and 5 mL/g TS for CP314 and C2064. This corresponds to reductions of 83–88% for lime, 97% for CP314 and 94–97% for C2064. The volume of settled sludge increased with higher dosages by −17–78% for lime, −38–122% for CP314 and −4–156% for C2064. Reasons for this are likely the increased settling of TSS and addition of TSS as lime, which increases the sludge mass and the floc size formed by polymers.[14] COD of the supernatant was only measured for one repetition and reduced from 1.9 g/l to 0.5–0.9 g/l for lime, 0.3–0.7 g/l for CP314 and 0.5–1.0 g/l for C2064, with lower concentrations at higher dosages. For polymer dosages exceeding 8 mL/g TS for CP314 and 5 mL/g TS for C2064, large flocs with poor settling were formed. This overdosing decreased settling and increased COD of the supernatant and the settled sludge volume compared to results at optimal dosage.

### 3.3. Dewatering

**Figure 3.**
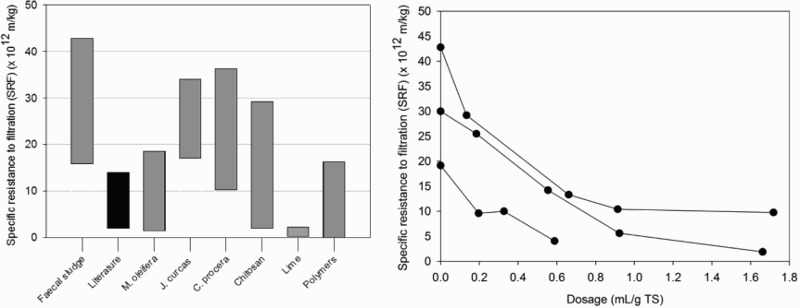
SRF results of unconditioned FS compared to FS conditioned with different conditioners, and SRF results of wastewater and drinking water sludge found in the literature (left). SRF results of FS conditioned with chitosan compared to unconditioned FS (right).

Figure 3 presents results of SRF experiments in this study compared to SRF of wastewater and drinking water sludge, as no references were found for SRF results with FS.[25,27,37] SRF of unconditioned FS was in the range of 15.9–42.8 × 10^12^ m/kg, which is poor compared to drinking and wastewater sludge, where SRF below 5 × 10^12^ m/kg is considered to be good.[30] In comparison, Ghebremichael and Hultman [37], Wai et al. [27] and Tat et al. [25] reported an SRF of 8.6–9.3 × 10^12^ m/kg, and 7.3–14.0 × 10^10^ m/kg and 2.0–9.1 × 10^11^ m/kg for drinking and wastewater sludge in Sweden and Malaysia, respectively.

As shown in Figure 3, conditioning with *M. oleifera* seeds and press cake reduced SRF. SRF decreased with higher dosages to 1.4–18.5 × 10^12^ m/kg and 1.4–12.9 × 10^12^ m/kg, respectively. Seeds and press cake produced similar results. Conditioning reduced SRF in the range of 30–95% and 51–96%, respectively. Dewatering was optimal at 10 mL/g TS. Conditioning of FS with *M. oleifera* seeds and press cake was more effective than previously observed with drinking water sludge with reductions in an SRF of 65% with dosages between 0.3 and 3.3 mL/g TS and an optimal dosage of around 1.3 mL/g TS.[37] In contrast to this study, an increase in conditioner dosage did not lead to a further reduction in SRF. Conditioning with a 10% stock solution reduced the SRF from 35.1 × 10^12^ m/kg to 12.1 × 10^12^ m/kg.

As shown in Figure 3, conditioning with chitosan decreased SRF. SRF decreased with higher dosages to 1.9–29.2 × 10^12^ m/kg corresponding to reductions of 15–94%. Dewatering was optimal at a dosage of 0.75 mL/g TS.

As with the previous results, in contrast to *M. oleifera* seeds and press cake, and chitosan, conditioning with *J. curcas* seeds and *C. procera* leaves did not consistently decrease SRF. Results were in the range of 17.0–34.0 × 10^12^ m/kg for *J. curcas* seeds and 10.3–36.3 × 10^12^ m/kg for *C. procera* leaves. This corresponds to a reduction in SRF of −46–25% for *J. curcas* seeds and -39–48% for *C. procera* leaves.

Conditioning with lime and commercially available polymers reduced SRF. SRF decreased with higher dosages to 0.2–2.2 × 10^12^ m/kg for lime, 0–12.1 × 10^12^ m/kg for CP314 and 0–13.0 × 10^12^ m/kg for C2064. This corresponds to reductions of 91–99% for lime, 37–100% for CP314 and 32–100% for C2064. Dewatering was optimal at a dosage of 0.3 g/g TS for lime and 5 mL/g TS for CP314 and C2064. In contrast to the other conditioners used in this study, high SRF values measured with conditioning by polymers did not appear to correlate to an insufficient dosage, but to the dissolution of flocs due to hydraulic disturbances when collecting settled sludge from Imhoff cones for SRF experiments. This has been reported for wastewater sludge where dewatering decreases with reduction in floc size.[15,37]

### 3.4. Comparison of conditioners

Lime and commercially available polymers were used in this study as a metric for comparison. Results from settling and dewatering experiments at optimal dosage are summarized in Table 3. As *M. oleifera* seeds and press cake, and lime increase the sludge mass and SRF is a function of sludge mass, absolute comparison of the results is limited. Still, the results of this study demonstrate that *M. oleifera* seeds and press cake and chitosan can have a similar performance (i.e. reduction in TSS in the supernatant and SRF) as lime and the commercially available polymers. Maximum reductions in TSS and SRF for *M. oleifera* seeds and press cake were within the range of results for lime and polymers, whereas maximum reductions with chitosan were 4–7% and 4–5% lower, respectively. Based on the lower performance and high variability, *J. curcas* seeds and *C. procera* appear to be unsuitable conditioners for FS and were not further considered in this study.

**Table 3.** Settling and dewatering results of conditioners at optimal dosage as determined in this study. *J. curcas* seeds and *C. procera* leaves are not reported, as their feasibility was ruled out based on their poor settling and dewatering performance.

	*M. oleifera* seeds and press cake	Chitosan	Lime	CP314	C2064
*Concentration*	5%	0.5%	–	0.5%	1%
*Settling*					
Optimal dosage	6–8 mL/g TS 300–400 kg^a^/t TS	0.3–0.6 mL/g TS 1.5–3.0 kg^b^/t TS	0.7–0.8 g/g TS 700–800 kg/t TS	125 mL; <5 mL/g TS 25 kg^c^/t TS	60 mL; <5 mL/g TS 50 kg^c^/t TS
TSS	<0.2 g/l	<0.3 g/l	<0.2 g/l	<0.2 g/l	<0.1 g/l
TSS reduction	81–95%	88–90%	83–88%	97%	94%
*Dewatering*					
Optimal dosage	10 mL/g TS 500 kg/t TS	0.75 mL/g TS 3.75 kg/t TS	0.3 g/g TS 300 kg/t TS	5 mL/g TS 25 kg/t TS	5 mL/g TS 50 kg/t TS
SRF reduction	69–93%	75–92%	91–95%	96–97%	97–100%

^a^Dry and shelled *M. oleifera* seeds.

^b^Dry chitosan.

^c^Stock solution provided by the manufacturer.

However, as shown in Table 3, in comparison to chitosan and the commercially available polymers *M. oleifera* seeds and press cake are much less efficient. They require 300–500 kg/t TS dry and shelled *M. oleifera* seeds or press cake. In comparison, CP314 and C2064 require 25 and 50 kg/t TS, respectively. In comparison, chitosan is more efficient requiring 2.0–3.75 kg/t TS dry chitosan. Considering settling and dewatering performance, required conditioner dosage per mass of TS, and local availability of resources, chitosan is the optimal conditioner for FS of those evaluated in this study.

In this study, an overdose effect was observed for polymers at high dosages. This effect was much less pronounced for chitosan and was absent for the other conditioners used in this study. This has important implications for full-scale treatment, as in practice the high variability of TS in FS (Table 2 and [31]) will make exact dosing based on solids concentrations difficult, and could easily lead to an overdose effect and reduced settling. Therefore, optimal usage of these conditioners would require measures such as a holding tank to homogenize the characteristics of FS. For example in Japan, FSTPs commonly have holding tanks with a capacity three times the daily influent volume.[38]

Identified optimal dosages were comparable to those recommended for wastewater sludge treatment of 0.02–0.2 mL/g TS for chitosan, 0.1–0.4 g/g TS for lime and 2 mL/g TS for C2064.[39–41] For chitosan, optimal dosages recommended by the manufacturer suggest that further optimization to reduce the dosage is possible. Optimal dosages were not available for CP314 and *M. oleifera* seeds and press cake as previous studies do not report dosages as a function of TS. The variability in conditioning results at similar dosages and differences observed between this study and manufacturer’s directions can be explained by the variability of FS and different characteristics of FS compared to wastewater sludge. The results indicate that optimal dosages for wastewater sludge conditioners are potentially similar for FS; however, in this study only two conditioners with similar molecular weights and structures were assessed with FS mostly from septic tanks. Therefore, other conditioners and sludges (e.g. public toilet FS, pit latrine FS) would need to be investigated prior to implementation.

The scalability of results from laboratory-scale Imhoff cone and SRF experiments is not certain for settling-thickening tanks and drying beds.[14,42] For example, [19] observed settling efficiencies of 60% in full-scale treatment due to hydraulic disturbance, which is lower than 69–95% in this study. Also, dewatering on drying beds is due to gravity, in contrast to SRF experiments where it is by vacuum. Due to this uncertainty, further bench-scale experiments with settling and dewatering columns were conducted with chitosan, lime, CP314 and C2064 to evaluate whether Imhoff cone and SRF experiments are replicable.

### 3.5. Settling and dewatering columns

In settling columns with unconditioned FS no clear solid–liquid interface between the supernatant and settled sludge was visible, whereas conditioned FS particles settled as a sludge blanket with a clear solid–liquid interface. The absence of a clear solid–liquid interface in unconditioned FS meant that settling velocity could not be determined. The settling efficiency of unconditioned FS in columns was greater than that in Imhoff cones. TSS in the supernatant of unconditioned FS reduced to 0.4–0.5 g/l and had TSS concentrations 86–90% of unconditioned FS before settling. In dewatering columns, the rate of percolation reduced when 90% of the total leachate volume had percolated. For unconditioned FS, 90% of the leachate percolated within 18–79 hours.

Results from settling of conditioned FS in columns confirmed the results from Imhoff cone experiments. TSS in the supernatant reduced to 0.2–0.4 g/l for chitosan which corresponds to reductions of 22–59% compared to unconditioned FS. Settling velocities were between 11 and 12 cm/min. In comparison to lime and polymers, TSS in the supernatant was reduced to 0.2 g/l for lime, 0.2 g/l for CP314 and 0.1–0.2 g/l for C2064. This corresponds to reductions compared to unconditioned FS of 43–64% for lime, 43–58% for CP314 and 59–81% for C2064. Settling velocities were in the range of 9–24 cm/min for lime, 13–14 cm/min for CP314 and 23 cm/min for C2064.

However, this means that TSS reductions in columns were in the range of 31–66% lower for chitosan, 24–40% for lime, 39–54% for CP314 and 13–35% for C2064 compared to results from Imhoff cone experiments shown in Table 3. This could be attributed to the lower initial TSS concentration and higher settling efficiency of unconditioned FS in settling columns compared to Imhoff cones. This can be explained by the prolonged settling time and the greater diameter of settling columns, as wall effects can influence settling in Imhoff cones.[14] These results indicate that Imhoff cone experiments might not be indicative for the absolute settling performance in settling–thickening but for comparison of different conditioners. Settling results within the same repetition had good replications with differences in settling velocity of 2–4 cm/min. In contrast, replication of settling with the same conditioner dosage and FS used in different repetitions had a high variability.

Results from dewatering of conditioned FS in columns also confirmed the results from SRF experiments. 90% of leachate percolated within 3–31 hours for chitosan. This corresponds to a reduction in dewatering time of 57–82% for chitosan. In comparison, 90% of leachate percolated within 2–13 hours for lime, 3–32 hours for CP314 and 2–6 hours for C2064. This corresponds to a reduction in dewatering time of 73–86% for lime, 59–83% for CP314 and 88–97% for C2064. This means that reductions in dewatering time in columns were in the range of 10–18% lower for chitosan, 9–18% for lime, 14–37% for CP314 and 3–9% for C2064 compared to results from SRF experiments shown in Table 3. However, the results demonstrate that SRF can be indicative for the relative increase in dewatering by FS conditioning on drying beds. Results varied between repetitions but this is to be expected due to different solid and hydraulic filter loading rates based on TS concentrations.[12]

### 3.6. Implications for faecal sludge treatment and resource recovery

In this study, optimal dosages were not the same for settling and dewatering. In addition, in full-scale treatment, hydraulic disturbances such as pumping sludge from settling–thickening tanks to drying beds can destroy flocs, thereby reducing improved dewatering. Hence, the location of dosing should be dependent on the treatment goal. For improved settling of TSS, FS should be conditioned prior to settling-thickening tanks, whereas for dewatering of FS on drying beds, the location should be settled sludge prior to loading drying beds.

The results indicate that conditioning does not increase the settling velocity, but does enhance the removal of TSS. This has important implications when removal of TSS is the treatment goal, for example if treatment of settling–thickening tank effluent is overloaded. In these cases, conditioning of FS could potentially be used to increase performance versus capital costs of constructing additional treatment capacity. However, as reported above, the influence of conditioning on physical and biochemical parameters needs to be carefully monitored, as *M. oleifera* seeds and press cake can increase COD and nutrient concentrations in the effluent.[22]

Conditioning increased dewatering of FS, thereby showing great potential to increase treatment capacities of existing treatment plants and/or reducing the required land area of future plants. It could also increase efficiencies of mechanical dewatering devices. The actual reduction in required drying bed area will depend on treatment goals, and the required dryness for end-use of treatment products. For example, in a pilot-scale study in Dakar, the required time for leachate to drain from drying beds was on average three days for a loading rate of 100 kg TS/m^2^*year, and seven days for a loading rate of 150 kg TS/m^2^*year [12] at which sludge has a dryness sufficient for use a soil conditioner or co-composting.[43,44] In contrast, use of FS for a dry combustion fuel requires dryness of 90%TS, and takes 16 days longer for a loading rate of 100 kg TS/m^2^*year, and 19 days longer for a loading rate of 150 kg TS/m^2^*year.[12,45] This means that the use of chitosan, lime or polymers could reduce dewatering times by 57–97% for use as a soil conditioner, or 9–15% and 15–26% for use as solid fuel at loading rates of 100 and 150 kg TS/m^2^*a, respectively.

In addition, other ramifications of conditioning on resource recovery have to be considered. For example, *M. oleifera* seeds and press cake and chitosan are organic and could increase beneficial properties of treatment end-products, such as nutrient and calorific value.[36] In contrast, lime is inorganic and would reduce the calorific value and fuel potential. However, lime conditioning has the advantage that it stabilizes the sludge and contributes to pathogen inactivation by raising the pH. In this study, conditioning with lime increased the pH to 12 for at least 12 hours. This means that a considerable inactivation of pathogens can be expected over time. For example,[46] reported an inactivation for Ascaris eggs greater than 99% after storage durations of 105–117 days for FS from pit latrines conditioned with lime dosages between 10 and 11 g/kg FS.

### 3.7. Availability and costs

The results of this study demonstrate that by improving settling and dewatering, FS conditioning can increase the effluent quality from settling–thickening tanks and reduce required space for dewatering, thereby increasing capacities of FSTPs. These benefits need to be balanced with increased operational, maintenance and capital costs, and implications for resource recovery. Whereas chemical grade lime and polymers are available in Europe and North America, they would need to be imported to sub-Saharan Africa. Maintaining a consistent supply of products for FS treatment can be a challenge due to high costs and long shipping and custom clearance time.[21] In contrast, *M. oleifera* seeds and press cake, and chitosan could be produced locally with available resources, which could decrease product costs and increase security of supply.

Operating experience of three functioning FSTPs in Dakar was used to evaluate the availability of locally available conditioners for FS treatment. An estimated 1500 m^3^/day of FS is delivered to the FSTPs, or 13.8 t TS/day based on the average concentration of 9.2 g TS/l (Table 2).[47] For optimal settling and dewatering (Table 3) this would require 5.5–6.9 t of *M. oleifera* seeds or press cake, and 0.021–0.052 t of dry chitosan.

Although *M. oleifera* trees are pervasive in Senegal, currently, insufficient quantities of *M. oleifera seeds* or press cake are available for conditioning of FS. Only one commercial source was identified, extracting oil from *M. oleifera* seeds to produce two to five tonnes of *M. oleifera* press cake per year. In line with [48], *M. oleifera* press cake from oil extraction also appears to be the most economic source for conditioning, as *M. oleifera* seeds are expensive. In Senegal, the identified company pays approximately 1000 USD/t *M. oleifera* seeds, which translates to 1400 USD/t conditioner (dried and shelled *M. oleifera* seeds).[49] Due to the low quantities available, *M. oleifera* seeds and press cake currently appear to have a low potential for FS conditioning. However, in the future, increased use of *M. oleifera* seeds, for example, for production of biofuels, [50] and purification of the protein in *M. oleifera* seeds [51,52] could increase the quantities available for conditioners and reduce the required conditioner dosage.

In this study, chemical grade chitosan was used; however, production of chitosan from crustacean shells such as shrimp is feasible in low-income countries.[40] Shrimp shells can be used to produce 2–3 wt% chitosan.[40] For example, the 800–900 t of shrimp processed for export in Senegal in 2012 and 2013 could produce 16–24 t of chitosan.[53] Competing demand for shrimp shells is low, and chitosan from this source could be considered financially viable if sold for 13,000–14,000 USD/t.[40] In comparison, the global market price of chitosan is around 25,000–30,000 USD/t, and lime and polymers including transport to Dakar would be 265 and 2850 USD/t, respectively.[39,54,55]

Cambérène FSTP in Dakar was used to evaluate additional treatment costs for FS conditioning. As shown in Table 4, although chitosan has much higher units cost compared to all other conditioners, due to the lower required dosage treatment costs it would only increase operational costs by 10–26% for locally produced chitosan, or 21–52% for imported chitosan, in comparison to 40–107% for lime and 36–72% for polymers.[47]

**Table 4.** Estimates for additional treatment costs at Cambérène FSTP for the conditioners assessed in this study. The calculation is based on treatment costs and a treatment capacity of 94,111 m^3^ FS included in [51], and a TS concentration of 9.2 kg/m^3^ (see Table 2).

	Unit	Chitosan	Lime	CP314	C2064
*Availability*					
Required dosage	t/year	1.30–3.25	260–693	22	43
Conditioner cost	USD/t	13,500 27,500	265	2850	2850
*Costs*					
Treatment costs	USD/day	171,180	171,180	171,180	171,180
Additional treatment cost	USD/day	17,550–43,875 35,750–89,275	68,900–183,645	61,690	122,550
Increase in treatment costs	%	10–26% 21–52%	40–107	36	72

## 4. Conclusions

The results of this study indicate that conditioning could be implemented for increased settling and dewatering of FS, thereby increasing treatment capacity or reducing required land area for FSTPs in urban areas. Findings include the following:

- Dosages for conditioning of wastewater sludge appear to be transferable to septic tank FS.
- FS conditioners which could be produced with locally available resources (i.e. *M. oleifera* seeds and press cake, and chitosan) appear to be as effective as commercially available wastewater conditioners.
- Prior to full-scale implementation, conditioning with other types of FS (e.g. public toilet FS, pit latrine FS) needs to be investigated.
- Use of conditioners increases operation costs of FSTPs, but can offset capital costs required to increase capacity or for construction of new FSTPs.
- Least expensive options for conditioning will depend on local availability and markets. In the case of Dakar, Senegal, production of chitosan from shrimp waste could be half as expensive as polymers.

Supplementary_Material.docx

## Nomenclature

%TS: percent total solids
cm: centimetre
g/g TS: gram per gram total solids
g/kg FS: gram per kilogram faecal sludge
kg TS/m^2^*year: kilogram total solids per square metre per year
kg/l: kilogram per litre
kPa: kilopascal
m^3^/d: cubicmetre per day
mm: millimetre
mL/g TS: millilitre per gram total solids
USD/t: United States Dollar per tonne
vol.: volume
wt.: weight
wt%: weight percent
t: tonne
