# Supplementary material for: Locally produced natural conditioners for dewatering of faecal sludge
Source: Environ Technol. 2016 Apr 18;37(21):2802–14. doi: 10.1080/09593330.2016.1165293 (PMC5020332; doi:10.1080/09593330.2016.1165293)
Supplement: Supplementary_Material.docx [file tent_a_1165293_sm0257.docx]

**Supplementary information**

**Locally produced natural conditioners for dewatering of faecal sludge**

Moritz Gold^1^ (corresponding author), Pauline Dayer^2^, Marie Christine Amie Sene Faye^3^, Guillaume Clair^4^, Alsane Seck^5^, Seydou Niang^6^, Eberhard Morgenroth^1,2^, Linda Strande^1^

^1^Eawag: Swiss Federal Institute of Aquatic Science and Technology

8600 Duebendorf, Switzerland

[moritz.gold@eawag.ch](mailto:moritz.gold@eawag.ch), +41 58 765 50 15

^2^ETH Zürich, Institute of Environmental Engineering

8093 Zürich, Switzerland

^3^Faculty of Medicine, Pharmacy and Odontology, Department of Pharmacy

Cheikh Anta Diop University of Dakar

B.P 5005, Dakar-Fann, Senegal

^4^Department of Water Engineering, Polytech Nice-Sophia

B.P 145, 06903 Sophia Antipolis, France

^5^Institute of Environmental Sciences (ISE), Faculty of Sciences and Technics

Cheikh Anta Diop University of Dakar

B.P 5005, Dakar-Fann, Senegal

^6^Laboratory of Wastewater Treatment, Fundamental Institute of Black Africa (IFAN)

Cheikh Anta Diop University of Dakar

B.P 206, Dakar-Fann, Senegal

|  | **Units** | **Unconditioned**  **FS (control)** | ***M. oleifera***  **seeds** | ***M. oleifera***  **press cake** | ***J. curcas***  **seeds** | ***C. procera***  **leaves** | **Lime** | **Chitosan** | **CP314** | **C2064** |
| --- | --- | --- | --- | --- | --- | --- | --- | --- | --- | --- |
| Conditioner  concentration |  | - | 5%  (wt./vol.) | 5%  (wt./vol.) | 5%  (wt./vol.) | 5%  (wt./vol.) | - | 0.5%  (wt./vol.) | 0.5%  (wt./vol.) | 1%  (wt./vol.) |
| **Laboratory experiments** | | |  |  |  |  |  |  |  |  |
| ***Settling*** |  |  |  |  |  |  |  |  |  |  |
| Repetitions | - | 10 | 8 | 3 | 4 | 4 | 3 | 3 | 3 | 3 |
| Dosages | ml/g TS  g/g TS | - | 3.9‑46.4 | 3.8-25.1 | 2-40 | 0.0085‑14.1 | 0.3‑ 2.4 | 0.07-1.7 | 2.9‑27.7 | 1.3‑18.5 |
| TSS supernatant | g/l | 0.7-3.6 | 0.02-0.5 | 0.01-0.4 | 0.5-2.5 | 0.6‑3.3 | 0.08-0.4 | 0.07-0.45 | 0.01-0.4 | 0.02-0.7 |
| TSS supernatant  - reduction | % | - | 35‑98 | 89-99 | -53-133 | -57-175 | 63-93 | 46-92 | 66-99 | 56-98 |
| Settled sludge volume  - increase | % | - | 52-310% | 61-194% | 30-155 | -11-48 | -17‑78 | 6‑78 | -38-122 | -4-156 |
| Floc size | - | - | < 1 mm | < 1 mm | - | - | < 1 mm | approx. half compared to  commercial conditioner | in the order of a few mm to a few cm | |
| Overdose effect | - | - | No | No | No | No | No | Yes | Yes  > 8 ml/g TS | Yes  > 5 ml/g TS |
| ***Dewatering*** |  |  |  |  |  |  |  |  |  |  |
| Repetitions | - | 10 | 8 | 3 | 4 | 4 | 3 | 3 | 3 | 3 |
| SRF | x 10^12^ m/kg | 15.9-42.8 | 1.4-18.5 | 1.4-12.9 | 17.0-34.0 | 10.3-36.3 | 0.2‑2.2 | 1.9‑29.2 | 0-12.1 | 0-13.0 |
| SRF reduction | % | - | 30-95 | 51-96 | -46-25 | -39-48 | 91-99 | 15-94 | 37-100 | 32-100 |
| **Bench-scale experiments** |  |  |  |  |  |  |  |  |  |  |
| ***Settling*** |  |  |  |  |  |  |  |  |  |  |
| Repetitions | - | 3 | - | - | - | - | 3 | 3 | 3 | 3 |
| Dosage | ml/g TS  g/g TS | - | - | - | - | - | 0.9-1.9 | 0.9-1.6 | 3.6-3.9 | 3.7-3.9 |
| TSS | g/l | 0.4-0.5 g/l | - | - | - | - | 0.2 | 0.2-0.4 | 0.2 | 0.1-0.2 |
| TSS reduction | % | - | - | - | - | - | 43-64 | 22-59 | 43-58 | 59-81 |
| Settling velocity | cm/min | - | - | - | - | - | 9-24 | 11-12 | 13-14 | 23 |
| ***Dewatering*** |  |  |  |  |  |  |  |  |  |  |
| Repetitions | - | 3 | - | - | - | - | 3 | 3 | 3 | 3 |
| Solid loading rate | kg TS/m^2^ | 2.5‑5.7 | - | - | - | - | 9.5-13.1 | 2.8-5.8 | 2.8-5.8 | 2.9‑6.0 |
| Hydraulic loading rate | cm | 9-19 |  |  |  |  | 12-20 | 13-20 | 9-16 | 11-22 |
| 90% percolation time | hours | 18-79 | - | - | - | - | 2-13 | 3-31 | 3-32 | 2-6 |
| 90% percolation time  - reduction | % | - | - | - | - | - | 73-86 | 57-82 | 59-83 | 88-97 |
